# Supplementary material for: Psychological Responses to Home-Working Practices: A Network Analysis of Relationships with Health Behaviour and Wellbeing
Source: Behav Sci (Basel). 2024 Nov 5;14(11):1039. doi: 10.3390/bs14111039 (PMC11591258; doi:10.3390/bs14111039)
Supplement: Supplementary file 1 [file behavsci-14-01039-s001.zip › behavsci-3114337-supplementary.pdf]

Supplementary materials

Supplementary Table 1 (ST1): *Item generation study: items, factor loadings and reliability (N = 240)*

*Latent Factor (prefix)**	*Item (factor loading**)*	*α****	*SBf ****
1. Pressure to work at the same time as colleagues (2)	My colleagues expect me to work the same amount of hours as they do (.775)	.83	
	I feel I have to work at the same time as my colleagues (.754)		
	I have no choice but to work for the same amount of hours as my colleagues (.665)		
2. Perceived home-working autonomy (2)	I am free to choose when I am online during the working day (.73)	.81	
	I am free to complete my work tasks at whatever time I wish (.714)		
	I can choose how many hours to work each day (.671)		
3. Pressure to attend meetings (2)	My colleagues expect me to attend all work-related meetings (.715)	.76	
	I feel pressured to attend all work-related meetings (.615)		
	I am free to decide how many relevant work-related meetings I attend (.556)		
4. Ability to switch off (3)	I think about work long after I have finished the working day (.718)		0.91
	I am able to disconnect from work after I have finished the working day (.698)		
5. Freedom to transition between home & working environments (4)	I am able to leave the space that I was working in (.935)		0.95
	I am able to move to a space that I don't use for work (.867)		
6. Ability to transform workspaces (2)	It is difficult for me to pack away my work-related items (e.g. computer, documents etc.) when I finish the working day (.608)	.83	

*Latent Factor (prefix)**	*Item (factor loading**)*	*α****	*SBf****
	I am free to pack away my work-related items (e.g. computer, documents etc.) when I finish the working day (.599)		
	I have no choice but to leave my work-related items (e.g. computer, documents etc.) set up when I finish the working day (.475)		
7. Workload manageability (2)	I have enough time in the day to deal with my workload (.723)		0.76
	It is difficult for me to take breaks (.529)		
8. Perceived excess of daily work meetings (2)	I feel that the amount of meetings I have is excessive (.987)		0.96
	I feel that I attend too many meetings (.934)		
9. Video on / off pressure (2)	My colleagues expect me to have my video on during video calls (.778)	.84	
	I feel that I should have my video on during video calls (.706)		
	I am free to have my video off during video calls (.875)		
10. Work-day planning & organization (3)	I make sure to plan out each working day (.931)		0.95
	I plan each day to make sure I get all my work done (.866)		

Note. *Prefix 1 = *'When working from home…'* (measured on frequency scale). Prefix 2 = '*When working from home…'* (agreement scale). Prefix 3 = *'On days when I work from home…'* (frequency scale). Prefix 4 = *'When I finish a home-working day…'* (agreement scale)

**Values in item parentheses are the factor loadings derived from the item development process of the previous study.

*** α = Cronbach's alpha, reported only for factors with three items or more. For factors with two items, reliability was modified using the Spearman-Brown formula (SBf) (Eisinga et al., 2013).

Supplementary Table 2 (ST2): *Main study: Participant Characteristics (N = 491)*

Demographic variable	*n*	%	M	SD
Age (years)	491	100	36.59	10.40
Gender				
Female	272	55.40		
Male	218	44.40		
Non-Binary	1	0.20		
Cohabitation				
Live with others	429	87.37		
Live on own	62	12.63		
Ethnicity				
White British - English, Welsh, Scottish, Northern Irish	428	87.12		
Asian/Asian British - Pakistani	13	2.65		
Black, African, Caribbean or Black British	12	2.44		
Asian/Asian British – Indian	11	2.24		
Asian/Asian British – Chinese	8	1.63		
Any other Asian background	6	1.22		
All other ethnicities*	14	2.65		
Occupational Industry				
Finance and Insurance	68	13.85		
Industry Other	64	13.03		
Education	61	12.42		
Government and Public Administration	52	10.59		
Health Care and Social Assistance	46	9.37		
Tech and Creative	40	8.15		
Infrastructure and Utilities	31	6.31		
Information Services and Data Processing	30	6.11		
Media and Entertainment	28	5.70		
Manufacturing	22	4.48		
Telecommunications	18	3.67		
Legal and Technical Services	17	3.46		
Retail and Wholesale	14	2.85		
Days worked per week	491	100	5.02	0.40
Job tenure (years)	491	100	5.28	4.98

Note. ‘Industry other’ was consolidated from: ‘Hotel and Food Services’ (1), ‘Military’ (1), ‘Other Industry – unspecified’ (51), ‘Religious’ (3),’Real Estate, Rental and Leasing’ (6), ‘Agriculture, Forestry, Fishing and Hunting’ (2); ‘All other ethnicities’ was consolidated from: ‘Bangladeshi’ (4), ‘Black Caribbean’ (3), ‘Black African’ (2), ‘Mixed White and Black African’ (2), ‘Mixed White and Asian’ (2), ‘Other Ethnic Group’ (1).

Supplementary Analysis: Socio-demographic differences in variable scores.

Given the presence of multivariate non-normality (see Supplementary Table 3), and prior to any data normalisation, a series of Kruskal-Wallis non-parametric tests were conducted to test for differences in the ten home-working response variables, ten health behaviour variables, and seven well-being variables across sociodemographic factors (gender, living situation, industry, and ethnicity). Due to the disproportionate representation of ethnicities, which was biased towards a predominant sample of White British (English, Welsh, Scottish, Northern Irish), the remaining ethnicities were consolidated into 'Ethnic other' to formulate a suitable comparison grouping for the analysis. Supplementary Table 3 presents the results of these analyses.

Gender

Dunn's post-hoc comparison tests with Bonferroni correction were conducted to inspect between-factor comparisons. Dunn's test indicated that males reported significantly higher pressure to work at the same time as colleagues (WFH 1) compared to females, p = .001. A significant difference was also observed for the ability to switch off from work (WFH 4), p = .005, with males reporting significantly lower scores (M = 3.78, SD = 1.70) than females (M = 4.26, SD = 1.64). Regarding the ability to transform workspaces, significant differences were found, p = .010, with males reporting higher scores (M = 5.30, SD = 1.84) than females (M = 4.88, SD = 1.94). For video on/off pressure (WFH 9), the difference between genders was marginally significant, p = .049, with males (M = 4.91, SD = 1.68) reporting higher scores than females (M = 4.59, SD = 1.72). For burnout, a significant difference was found, p = .003, with males reporting lower burnout levels compared to females. Similarly, a significant difference in stress was observed, p = .006, with males reporting lower stress levels compared to females. Finally, for work time physical activity, males reported significantly higher levels than females, p = .010.

Industry

Dunn's post-hoc test revealed a significant difference across industries in participant's reported freedom to transition between home and working environments, p = .04. The Retail and Wholesale sector reported significantly higher pressure (M = 4.10, SD = 2.14) compared to the Finance and Insurance sector (M = 3.54, SD = 1.50), p = .060. No other significant between-industry differences were found. A significant difference in work-life conflict was observed between industries, p = .04. Telecommunications employees reported the highest levels of work-life conflict (M = 47.22, SD = 35.11), significantly higher than those in Education (M = 25.41, SD = 21.01), p = .060, and Retail and Wholesale employees (M = 39.73, SD = 24.71), p = .020. Additionally, Telecommunications employees had significantly higher conflict than those in Finance and Insurance (M = 30.61, SD = 25.10), p = .020. Significant differences in self-reported health were found across industries, p = .03. Retail and Wholesale employees reported significantly higher health scores (M = 61.72, SD = 25.19) compared to Other industry employees (M = 47.22, SD = 24.08), p = .024. Cognitive stress levels also differed significantly by industry, p = .01. Telecommunications employees reported the highest levels of cognitive stress (M = 50.69, SD = 24.90), significantly higher than those in Information Services and Data Processing (M = 33.13, SD = 19.98), p = .012, and Health Care and Social Assistance (M = 37.91, SD = 23.84), p = .009. Finally, Sleep trouble varied significantly across industries, p = .04. Employees in Telecommunications

reported the highest levels of sleep trouble (M = 55.56, SD = 28.59), significantly more than those in Information Services and Data Processing (M = 28.33, SD = 20.28), *p* = .014. Retail and Wholesale employees (M = 50.89, SD = 25.93) also experienced more sleep trouble compared to Education (M = 38.11, SD = 25.58), *p* = .035.

Ethnicity

Dunn's post-hoc comparison test identified a significant difference across the ethnicity groups on pressure to work at the same time as colleagues, p = .013, with White participants reporting significantly higher pressure (M = 4.45, SD = 1.50) compared to those from other ethnic backgrounds (M = 3.98, SD = 1.57). Regarding the ability to switch off from work, White participants reported significantly higher scores (M = 5.14, SD = 1.91) compared to participants from other ethnic groups (M = 4.60, SD = 1.81), p = .004. For the perceived excess of daily work meetings, a significant difference was found, with participants from other ethnic groups reporting higher levels (M = 4.34, SD = 1.78) compared to White participants (M = 3.58, SD = 1.78), p = .001. In terms of work-life conflict, participants from other ethnic groups reported significantly higher levels of conflict (M = 37.60, SD = 29.11) compared to White participants (M = 27.06, SD = 23.96), p = .003.

Living situation

Dunn's post-hoc comparison tests highlighted significant differences in reported abilities to transform workspaces between participants living with others (M = 5.16, SD = 1.89) and those living alone (M = 4.44, SD = 1.93), p = .001. For break taking frequency, a significant difference was observed, p = .008, with those living with others taking more frequent breaks (M = 67.13, SD = 34.86) compared to those living alone (M = 55.89, SD = 32.97). Finally, a significant difference was found for work time sedentary behaviour, p = .010, where people living alone (M = 8.19, SD = 2.59) reported higher scores than those living with others (M = 7.56, SD = 2.13).

Supplementary Table 3 (ST3). *Main study: Kruskal-Wallis Test Results for Sociodemographic Differences Across Home-Working, Health Behavior, and Well-Being Variables (N = 491)*

V#	Dependent Variable	Demographic Factor	Chi-Squared (χ^2)	df	p-value
	Home-working responses		11.45	2	0.003**
1	Pressure to work at same time as colleagues	Gender	8.5	2	0.014**
4	Ability to switch off from work	Gender	44.35	28	0.026*
4	Ability to switch off from work	Industry	7.33	2	0.026*
6	Ability to transform workspaces	Gender	9.3	1	0.002**
6	Ability to transform workspaces	Living Situation	7.38	2	0.025*
9	Perceived 'video-on' pressure in meetings	Gender	6.03	2	0.049*
10	Workday planning and organisation	Gender	10.92	2	0.004**
14	Burnout	Gender	10.08	2	0.006**
15	Stress	Gender	23.02	13	0.042*
22	Walking	Ethnicity	5.75	1	0.016*
25	Break taking frequency	Living Situation	22.62	13	0.046*
25	Break taking frequency	Ethnicity	5.41	1	0.02*
26	Work time sedentary behaviour	Living Situation	7.49	2	0.024*
23	Work time physical activity	Gender	11.45	2	0.003**

Note. * $p<.05$, ** $P<.01$

Supplementary Table 4 (ST4): *Main study: Itemised and numbered breakdown of measured home-working responses, work-related wellbeing, health, health behaviour, and demographic items (N = 491)*

V#	Measure	Mean	Std. Deviation	Pre Npn Skewness	Pre Npn Kurtosis	Post Npn Skewness	Post Npn Kurtosis
	Home-working responses						
1	Pressure to work at same time as colleagues	4.60	1.50	-0.44	-0.48	-0.04	-0.36
2	Home-working autonomy	3.84	1.54	0.01	-0.77	-0.03	-0.36
3	Pressure to attend meetings	5.06	1.25	-0.62	0.27	-0.06	-0.44
4	Ability to switch off from work	4.27	1.48	-0.13	-0.79	0.03	-0.46
5	Freedom to transition between home & working environments	5.07	1.91	-0.83	-0.56	0.04	-0.39
6	Ability to transform workspaces	4.72	1.71	-0.41	-0.87	-0.27	-0.87
7	Daily workload manageability	4.39	1.52	-0.28	-0.83	-0.10	-0.40
8	Perceived excess of daily meetings	3.68	1.79	0.18	-1.18	0.06	-0.60
9	Video on / off pressure	4.05	1.68	-0.19	-1.00	-0.14	-0.61
10	Workday planning and organisation	4.18	1.48	-0.20	-0.64	-0.02	-0.42
	Work-related wellbeing						
11	Work life conflict	28.41	24.90	0.70	-0.21	0.31	-0.59
12	Cognitive stress	35.81	21.93	0.26	-0.61	0.10	-0.39
13	Job satisfaction	64.00	24.67	-0.81	0.16	-0.20	-0.15
14	Burnout	46.79	24.09	0.03	-0.69	0.00	-0.34
15	Stress	6.73	3.09	0.08	-0.25	0.00	-0.21
16	General wellbeing	21.38	3.45	0.73	0.80	0.01	-0.19
17	Isolation	5.24	1.87	0.44	-0.82	0.26	-0.88
	Health & Health behaviours						
18	Self-rated health	56.01	23.68	-0.14	-0.44	-0.06	-0.29
19	Sleep trouble	36.00	23.90	0.49	-0.49	0.09	-0.44
20	Vigorous exercise	677.31	1108.25	2.40	7.11	0.89	-0.29
21	Moderate exercise	273.18	484.95	2.53	6.93	0.94	-0.21
22	Walking	613.75	684.18	2.17	6.03	0.21	-0.52
23	Work time physical activity	2.91	1.77	0.52	-1.10	0.35	-0.71
24	Total sedentary behaviour	8.91	1.83	0.20	0.33	-0.07	-0.26
25	Break taking frequency	82.97	50.36	0.73	-0.44	-0.15	-0.66
26	Work time sedentary behaviour	7.64	2.20	1.82	7.55	0.02	-0.03
27	Snacking	11.54	7.75	0.59	-0.56	-0.07	-0.44
	Demographics						
28	Age	36.59	10.41	0.78	-0.12	0.01	-0.17
29	Job tenure	5.28	4.98	1.57	2.64	0.19	-0.45

Note. V# = Variable network number; Npn = Non-parametric transformation.

Supplementary Table 5 (ST5): *Main study: Bivariate correlation matrix for all observed variables, prior to regularisation (N = 491)*

	1.	2.	3.	4.	5.	6.	7.	8.	9.	10.	11.	12.	13.	14.	15.
2.	-0.54**														
3.	0.56**	-0.52**													
4.	-0.14**	0.06	-0.23**												
5.	-0.08	0.07	-0.13**	0.3**											
6.	-0.21**	0.09	-0.26**	0.41**	0.4**										
7.	-0.27**	0.24**	-0.34**	0.52**	0.25**	0.38**									
8.	0.21**	-0.2**	0.34**	-0.29**	-0.1*	-0.2**	-0.48**								
9.	0.27**	-0.2**	0.32**	-0.22**	-0.04	-0.18**	-0.27**	0.19**							
10.	-0.03	0.11*	-0.04	-0.04	0.1*	0.13**	-0.02	0.06	0.14**						
11.	0.26**	-0.22**	0.31**	-0.56**	-0.27**	-0.37**	-0.54**	0.36**	0.13**	-0.11*					
12.	0.15**	-0.09*	0.17**	-0.35**	-0.27**	-0.26**	-0.32**	0.17**	0.05	-0.22**	0.5**				
13.	-0.2**	0.21**	-0.17**	0.21**	0.15**	0.18**	0.27**	-0.13**	0	0.29**	-0.41**	-0.38**			
14.	0.17**	-0.14**	0.24**	-0.47**	-0.21**	-0.29**	-0.42**	0.26**	0.1*	-0.16**	0.61**	0.61**	-0.35**		
15.	0.17**	-0.17**	0.18**	-0.39**	-0.21**	-0.28**	-0.36**	0.2**	0.11*	-0.2**	0.54**	0.64**	-0.44**	0.62**	
16.	-0.17**	0.17**	-0.17**	0.33**	0.27**	0.21**	0.31**	-0.14**	-0.05	0.28**	-0.48**	-0.62**	0.57**	-0.54**	-0.66**
17.	0.18**	-0.11*	0.19**	-0.25**	-0.22**	-0.23**	-0.24**	0.09	0	-0.18**	0.44**	0.43**	-0.29**	0.45**	0.49**
18.	-0.11*	0.1*	-0.11*	0.22**	0.1*	0.2**	0.23**	-0.13**	-0.08	0.22**	-0.35**	-0.28**	0.27**	-0.41**	-0.37**
19.	0.05	-0.04	0.07	-0.37**	-0.19**	-0.23**	-0.3**	0.22**	0.03	-0.11*	0.41**	0.44**	-0.26**	0.5**	0.46**
20.	-0.01	0.05	-0.07	-0.08	-0.02	-0.06	0.05	-0.01	-0.04	0.05	-0.01	0.02	0	-0.07	-0.07
21.	-0.02	0.09	-0.07	-0.01	-0.04	0	-0.02	0.07	-0.03	0.07	0.03	0.08	-0.03	0.06	0.05
22.	-0.05	0.01	-0.03	-0.06	-0.07	-0.07	-0.04	0.06	-0.08	0.06	0.04	0.04	-0.05	-0.03	-0.01
23.	-0.05	0.16**	-0.17**	0.07	0.03	0.09	0.18**	-0.03	0	0.14**	-0.16**	-0.06	0.06	-0.15**	-0.14**
24.	-0.01	-0.08	0.02	0.07	0.01	0.04	0.1*	-0.06	-0.01	-0.18**	0	-0.01	-0.04	0.03	0.03
25.	0.18**	-0.2**	0.23**	-0.1*	-0.02	-0.1*	-0.19**	0.08	0.06	-0.02	0.11*	-0.02	-0.02	0.07	0.01
26.	0.11*	-0.1*	0.14**	-0.18**	-0.13**	-0.18**	-0.23**	0.12**	0.06	0	0.22**	0.08	-0.08	0.17**	0.13**
27.	0.01	0	0.06	-0.15**	-0.09*	-0.06	-0.08	-0.02	0.03	-0.14**	0.18**	0.22**	-0.1*	0.23**	0.2**
28.	-0.11*	0.16**	-0.13**	0.02	0.18**	0.02	0.03	0.04	0.05	0.16**	-0.1*	-0.22**	0.07	-0.16**	-0.1*
29.	-0.08	0.15**	-0.14**	0.01	0.1*	-0.02	0.04	0.03	0	0.15**	-0.05	-0.13**	0.1*	-0.09	-0.1*

	16.	17.	18.	19.	20.	21.	22.	23.	24.	25.	26.	27.	28.
17.	-0.49**												
18.	0.36**	-0.27**											
19.	-0.41**	0.33**	-0.29**										
20.	-0.06	0.02	0.17**	-0.02									
21.	-0.06	0.06	0.04	0.06	0.29**								
22.	-0.05	0.06	0.14**	0.02	0.17**	0.27**							
23.	0.12**	-0.12**	0.25**	-0.08	0.4**	0.2**	0.25**						
24.	-0.04	0.02	-0.11*	-0.04	-0.08	-0.08	-0.09	-0.11*					
25.	-0.04	0	-0.01	0.03	-0.1*	-0.1*	-0.03	-0.17**	0.09				
26.	-0.08	0.11*	-0.09*	0.05	-0.06	-0.09*	-0.09*	-0.2**	0.28**	0.2**			
27.	-0.17**	0.2**	-0.15**	0.09*	-0.06	0	-0.07	-0.07	0	-0.03	0.1*		
28.	0.18**	-0.18**	-0.02	0.04	-0.15**	-0.03	-0.01	-0.06	-0.06	-0.03	0	-0.04	
29.	0.16**	-0.09*	0.05	0.07	-0.05	0.04	0.02	0.02	-0.07	-0.09*	-0.04	-0.06	0.52**

Variables: 1 = Pressure to work at same time as colleagues; 2 = Homeworking autonomy; 3 = Pressure to attend meetings; 4 = Ability to switch off from work; 5 = Freedom to transition between home and working environments; 6 = Ability to transform workspaces; 7 = Daily workload manageability; 8 = Perceived excess of daily work meetings; 9 = Video on / off pressure; 10 = Work-day planning & organisation; 11 = Work life conflict; 12 = Cognitive stress; 13 = Job satisfaction; 14 = Burnout; 15 = Stress; 16 = General wellbeing; 17 = Isolation; 18 = Self-rated health; 19 = Sleep trouble; 20 = Vigorous exercise; 21 = Moderate exercise; 22 = Walking; 23 = Work time physical activity; 24 = Overall sedentary behaviour; 25 = Break taking; 26 = Work time sedentary behaviour; 27 = Snacking; 28 = Age; 29 = Job tenure.

Supplementary Figure 1 (SF1). *Main study: Standardised Z-scores for node centrality indices – Strength, Closeness, Betweenness, & Expected Influence.*

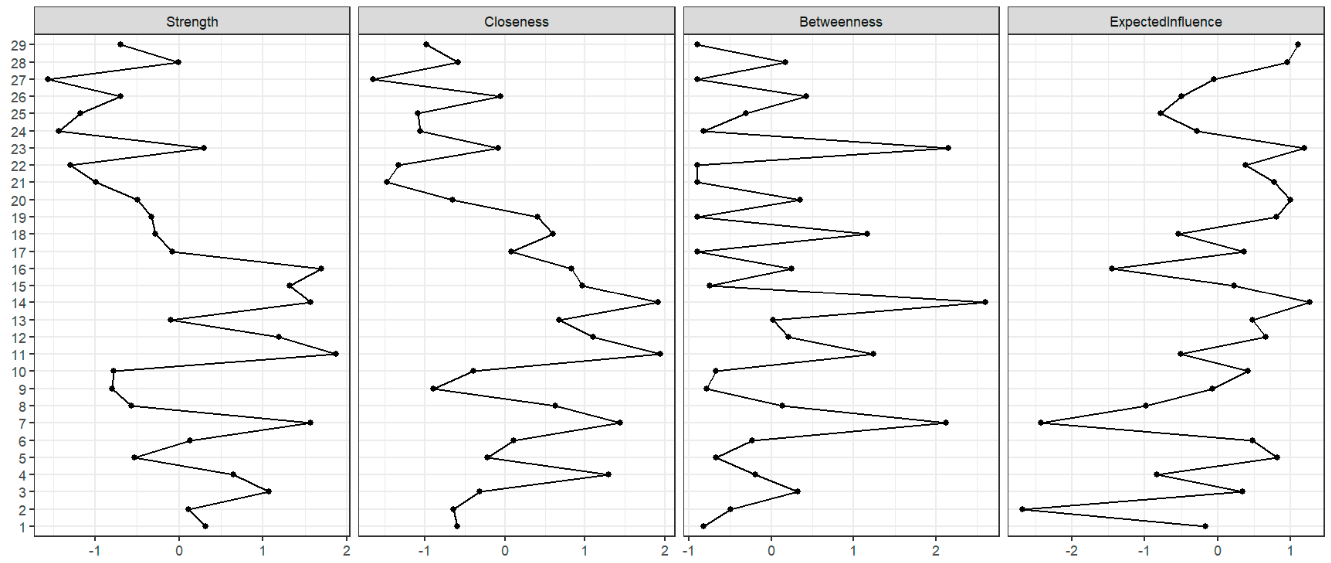

Supplementary Figure 2 (SF2): *Main study: Bootstrapped (2000) edge weight accuracy.*

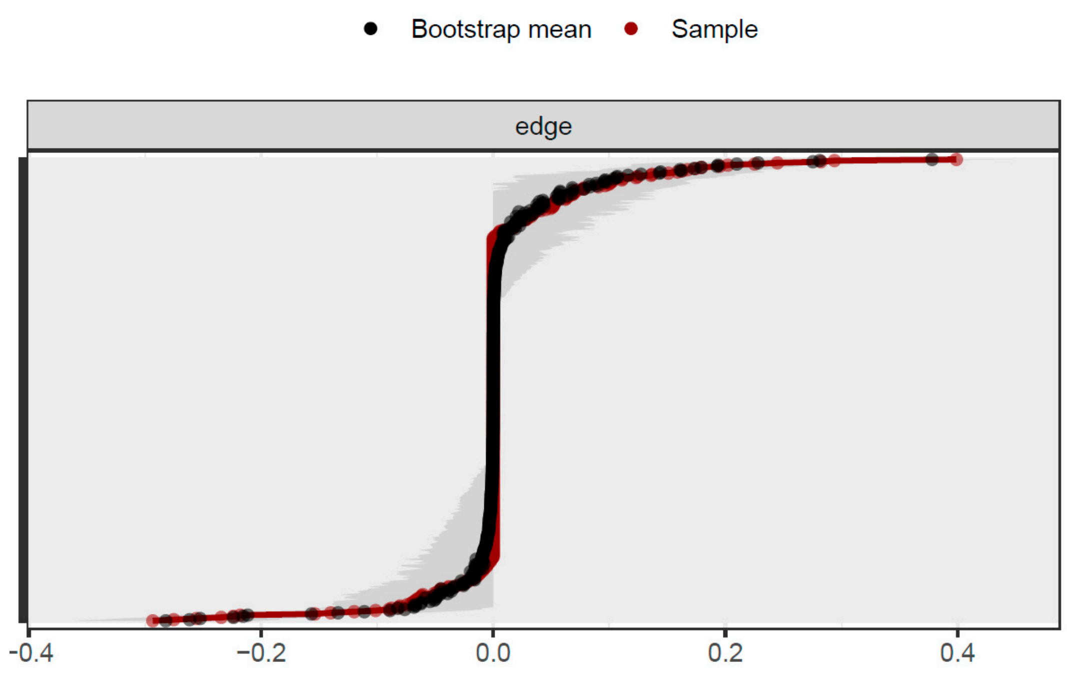

Supplementary Figure 3 (SF3): *Main study: Bootstrapped (2000) centrality stability: Strength, closeness, betweenness, expected influence.*

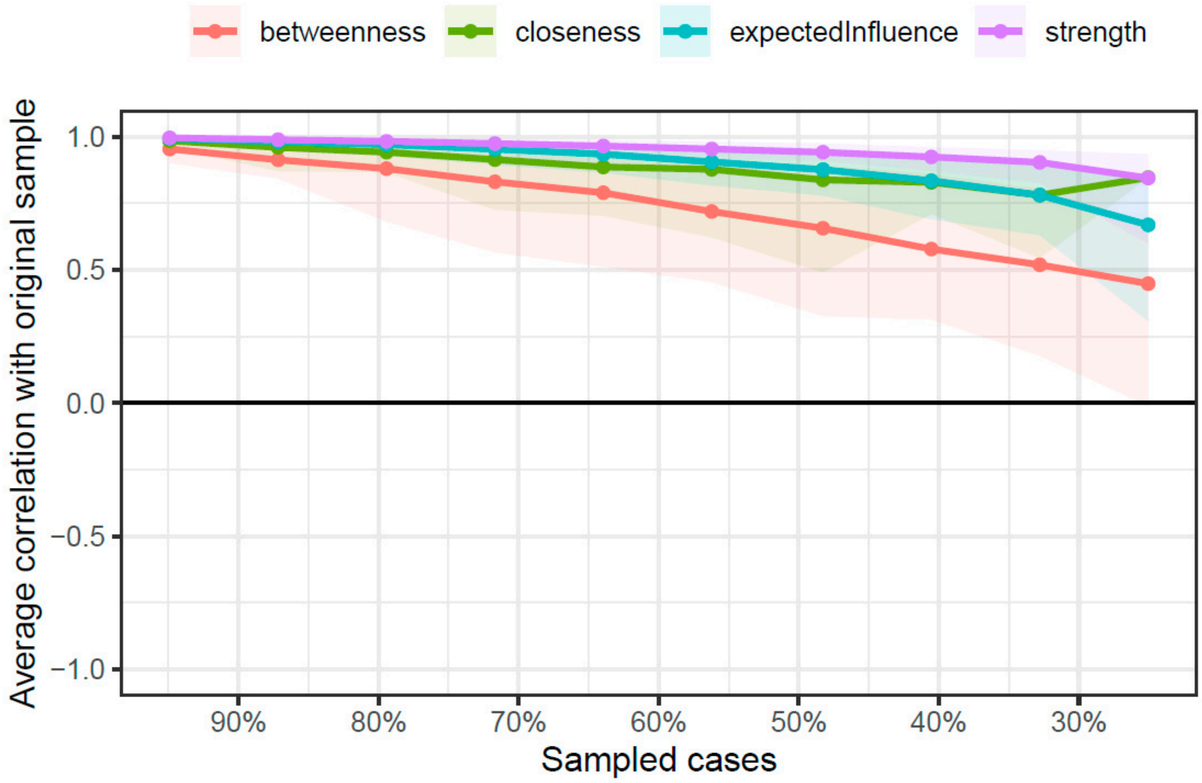

Note. Maximum drop proportions to retain correlation of 0.7 in at least 95% of the samples: Betweenness: 0.216, Closeness: 0.128, Expected Influence: 0.595, Strength: 0.672.

Supplementary Figure 4 (SF4): *Main study: Standardised Z-scores for Bridge node centrality: Bridge expected influence [1-step].*

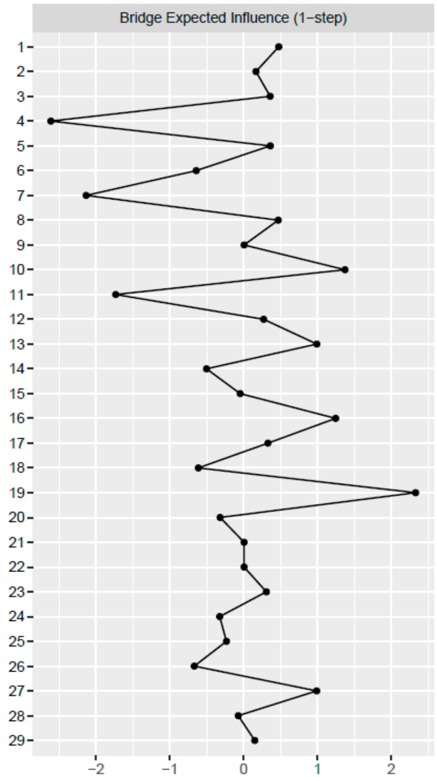

Supplementary Figure 5 (SF5): *Main study: Bootstrapped (2000) bridge node centrality stability: Bridge expected influence [1-step].*

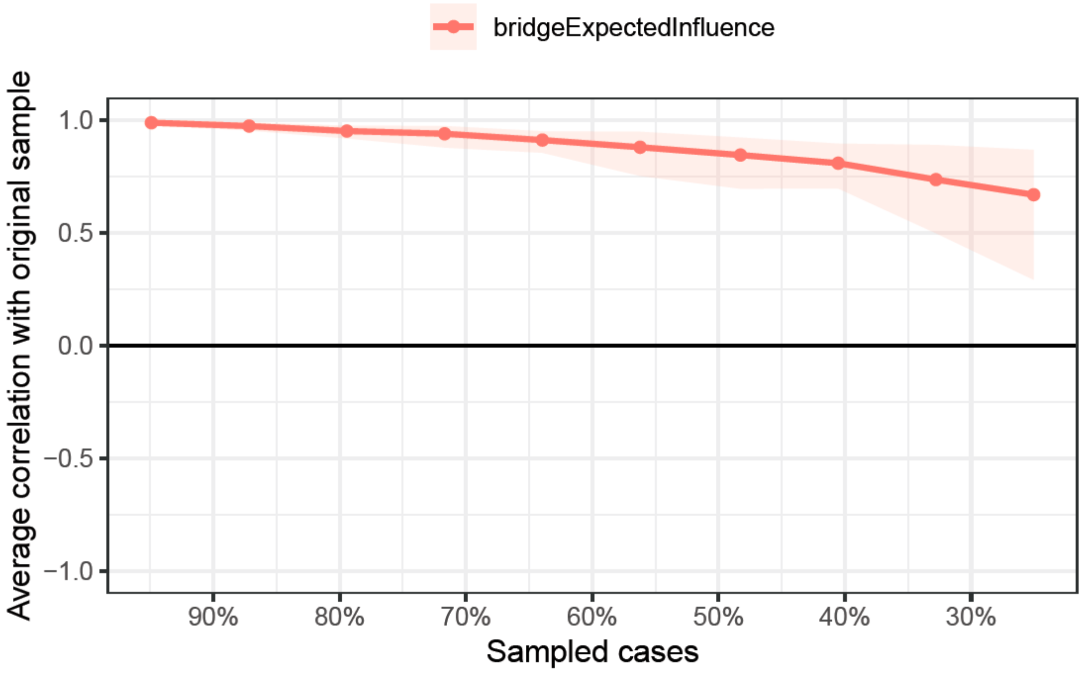

Note. Maximum drop proportions to retain correlation of 0.7 in at least 95% of the samples: Bridge expected influence [1-step]: 0.517

Supplementary Methods

Network accuracy and centrality stability

The accuracy of the estimated network and the stability of centrality indices were assessed through non-parametric bootstrapping methods via the boonet package (Epskamp et al., 2015). Estimated network accuracy was determined by calculating edge-weights in 2000 randomly allocated bootstrapped samples. Centrality stability was indicated via the case-drop bootstrap (2000), which estimated the maximum number of participants that could be dropped whilst retaining a correlation of ≥.70 between the original sample and iteratively smaller subsets of the sample (ranging between 90% - 30% of the sample, reduced by 10% iterations). CS coefficients (Correlational Stability) were calculated for each of the centrality indices to quantify the proportion of data that could be dropped to retain with 95% certainty a correlation of at least 0.7 with the original centrality value. CS coefficients indicate stability when above 0.5 (Epskamp et al., 2018).

Analyses

Centrality indices selection. Node characteristics can be examined via centrality indices, each aiming to assist in the exploration and discovery of influential nodes in the estimated network. Indices available to examine were node *strength*, *closeness*, *betweenness* (Hevey, 2018), and *expected influence* (Robinaugh et al., 2016). Due to the absolute value calculation, *strength* is limited to only accurately informing the centrality of positively associated edges. *Expected influence* addresses the *strength* indices limitation and informs us of the importance of when nodes both activate and deactivate one another. Our estimated network contains nodes where code reversal would be inappropriate, therefore the interpretation of *expected influence* was chosen over *strength* (Robinaugh et al., 2016). Nodes furthest away from the value of zero indicate a high expected influence, with the direction of association denoted by whether the expected influence is positive of negative. The remaining centrality indices (*betweenness & closeness*) were not included in the analyses due to demonstrated instability indicated by correlational Stability coefficients below 0.5 (see Supplementary Figure 3, above).

Supplementary Results

Network accuracy and centrality stability.

Calculated bootstrap confidence intervals for the relationships observed in the network network were small to moderate in width, indicating a good level of network accuracy (see Supplementary Figure 2). Of the observed centrality indices (see Supplementary Figure 3), only expected influence (CS [cor = 0.7] ≈ 0.67 demonstrated stability, whereas betweenness (CS [cor = 0.7] ≈ 0.28), and closeness (CS [cor = 0.7] ≈ 0.36) demonstrated instability with CS coefficients below 0.5 and were therefore treated with caution and excluded from the main interpretation (Epskamp & Fried, 2018; Hevey, 2018). CS coefficients were calculated to assess the stability of the considered centrality indices produced for the bridge analyses. Bridge expected influence (CS [cor = 0.7] ≈ 0.52) displayed acceptable stability, supporting its use as informative centrality indices.

Variable selection for shortest path analyses.

Based on the initial expected influence centrality values (see Supplementary Figure 1), the top indicator variables of wellbeing and health were selected to investigate the shortest pathways to homeworking experiences. From the indictors of wellbeing, general wellbeing

(V16, EI = -1.43) and burnout (V14, EI = 1.24) were selected. Among the health-related indicators, work-time physical activity (V23, EI: 1.19), vigorous exercise (V20, EI: 0.98), break taking (V25, EI: -0.88), sleep trouble (V19, EI: 0.83), and moderate exercise (V21, EI: 0.79) demonstrated the highest expected influence values. However, due to similar shortest pathways observed from variables 23, 20, and 21 to 25, only variables 23 (work-time physical activity), and 19 (sleep trouble) were chosen for focussed interpretation.

Supplementary References

Eisinga, R., te Grotenhuis, M., & Pelzer, B. (2013). The reliability of a two-item scale: Pearson, Cronbach, or Spearman-Brown? *International Journal of Public Health, 58*(4), 637–642. https://doi.org/10.1007/s00038-012-0416-3

Epskamp, S., Borsboom, D. & Fried, E.I. Estimating psychological networks and their accuracy: A tutorial paper. (2018). *Behavioral Research Methods, 50,* 195–212. https://doi.org/10.3758/s13428-017-0862-1

Epskamp, S., & Fried, E. I. (2018). A tutorial on regularized partial correlation networks. *Psychological methods*, *23*(4), 617–634. https://doi.org/10.1037/met0000167

Epskamp, S., & Fried, E. I. (2024). Package 'bootnet.' https://cran.r-project.org/web/packages/bootnet/index.html

Hevey D. (2018). Network analysis: a brief overview and tutorial. *Health psychology and behavioral medicine*, *6*(1), 301–328. https://doi.org/10.1080/21642850.2018.1521283

Robinaugh, D. J., Millner, A. J., & McNally, R. J. (2016). Identifying highly influential nodes in the complicated grief network. *Journal of abnormal psychology*, *125*(6), 747–757. https://doi.org/10.1037/abn0000181
